# A Mass‐Spectrometry‐Based Approach to Distinguish Annular and Specific Lipid Binding to Membrane Proteins

**DOI:** 10.1002/anie.201914411

**Published:** 2020-01-29

**Authors:** Jani Reddy Bolla, Robin A. Corey, Cagla Sahin, Joseph Gault, Alissa Hummer, Jonathan T. S. Hopper, David P. Lane, David Drew, Timothy M. Allison, Phillip J. Stansfeld, Carol V. Robinson, Michael Landreh

**Affiliations:** ^1^ Department of Chemistry University of Oxford South Parks Road Oxford OX1 3QZ UK; ^2^ Department of Biochemistry University of Oxford South Parks Road Oxford OX1 3QU UK; ^3^ Department of Microbiology, Tumor and Cell Biology Karolinska Institutet 17165 Solna Sweden; ^4^ Department of Biology University of Copenhagen Copenhagen N 2200 Denmark; ^5^ OMass Therapeutics The Oxford Science Park, The Schrödinger Building Kidlington OX4 4GE UK; ^6^ Department of Biochemistry and Biophysics Stockholm University 10691 Stockholm Sweden; ^7^ Biomolecular Interaction Centre and School of Physical and Chemical Sciences University of Canterbury Christchurch 8140 New Zealand; ^8^ School of Life Sciences & Department of Chemistry University of Warwick Coventry CV4 7AL UK

**Keywords:** lipid binding, membrane protein structure, molecular dynamics, native mass spectrometry

## Abstract

Membrane proteins engage in a variety of contacts with their surrounding lipids, but distinguishing between specifically bound lipids, and non‐specific, annular interactions is a challenging problem. Applying native mass spectrometry to three membrane protein complexes with different lipid‐binding properties, we explore the ability of detergents to compete with lipids bound in different environments. We show that lipids in annular positions on the presenilin homologue protease are subject to constant exchange with detergent. By contrast, detergent‐resistant lipids bound at the dimer interface in the leucine transporter show decreased k_off_ rates in molecular dynamics simulations. Turning to the lipid flippase MurJ, we find that addition of the natural substrate lipid‐II results in the formation of a 1:1 protein–lipid complex, where the lipid cannot be displaced by detergent from the highly protected active site. In summary, we distinguish annular from non‐annular lipids based on their exchange rates in solution.

## Graphical abstract

**Separating the wheat from the chaff**: Distinguishing specific and non‐specific lipid binding to membrane proteins is a major challenge. Non‐annular lipid interactions can be identified using a detergent competition assay and native mass spectrometry (MS). MD simulations reveal the location of specific lipid‐binding sites with slow *k*_off_ rates, confirming the MS data.

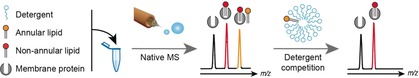

Membrane proteins rely on the lipid environment of the membrane to shape their structure and function, yet the nature of the underlying lipid interactions can be highly diverse.^1^, ^2^ Annular lipids form non‐specific contacts with the protein and exchange rapidly with the bulk lipids in the membrane. The function of annular lipids is to provide the amphiphilic environment required to keep the protein in a folded state, while non‐annular lipids have more defined functional roles, such as stabilization of oligomeric assemblies or binding to regulatory sites.^3^, ^4^ Due to the challenge of experimentally studying these proteins in the membrane, they are often solubilized by incubation with an excess amount of detergent in solution. This process breaks up the membrane and replaces protein–lipid interactions with protein–detergent ones. Interestingly, some detergent‐solubilized proteins retain bound lipids, which are sometimes observed in the electron density maps of X‐ray crystallography or cryo‐electron microscopy experiments.^5^ These co‐purified species are often specific lipids preferentially bound at non‐annular sites and provide functionally important interactions.^2^, ^3^ Therefore, by partitioning bound lipids into more detergent‐sensitive and more detergent‐resistant populations it may be possible to distinguish annular, rapidly exchanging lipids from non‐annular lipids that are involved in specific interactions. Here we ask 1) whether we can observe the exchange of annular lipids for detergent molecules, and 2) whether non‐annular lipids would exhibit different exchange properties. To answer these questions, we turned to native mass spectrometry (nMS), which enables the study of lipid binding to membrane proteins under a variety of solution conditions.^6^, ^7^ After release from the detergent micelle inside the mass spectrometer, it is possible to detect intact protein–lipid complexes and to measure their binding stoichiometry and stability in the gas phase.^8^, ^9^, ^10^, ^11^

We can follow lipid binding by adding lipids to detergent‐solubilized membrane proteins and measuring the molecular weight of the resulting complexes with nMS. We therefore hypothesized that it should also be possible to detect lipid‐to‐detergent exchange by measuring the fraction of bound exogenous lipids as a function of detergent concentration (Figure 1 a). We selected the 33 kDa presenilin homologue PSH from *Methanoculleus marisnigri,* because it is well established that it can be extracted from the membrane as a lipid‐free monomer and its activity is restored in detergent micelles with the addition of *Escherichia coli* lipids.^12^, ^13^ The addition of *E. coli* polar lipid extract to lipid‐free PSH in 0.2 % nonylglucoside (NG) and 0.03 % dimethyl‐dodecylamine *N*‐oxide (LDAO) resulted in a nMS spectrum with additional peaks for each charge state. The detergent conditions used in this study are summarized in Table S1 in the Supporting Information. The mass difference between the peaks corresponded to binding of up to three lipids per protein (Figure 1 b). Increasing the concentration of NG in a stepwise fashion from 0.2 % to 0.5 % led to a gradual removal of lipid adduct peaks, in line with the delipidating properties of NG^14^ (Figure 1 b). Together these results show that it is possible to follow lipid exchange with detergent in a concentration‐dependent manner. Since we added all three of the main *E. coli* lipids simultaneously, phosphatidyl‐ethanolamines (PE), phosphatidylglycerols (PG), and cardiolipins (CDL), we could observe that increasing the concentration of NG induces stepwise removal of all three lipids, indicating no difference in the extent of removal of each lipid with respect to detergent concentration; the different lipids were displaced at comparable rates. We therefore conclude that there is no marked preference for binding of a particular class of lipid to PSH (Figure 1 b and Supporting Information, Figure S1). The competition, observed experimentally for PSH, between lipids and detergent molecules is consistent with the annular lipid interactions predicted by molecular dynamics (MD) simulations of membrane proteins in mixed lipid–detergent micelles.^16^ Lipids added to solubilized PSH are therefore in exchange with the detergent when binding to the protein, and become more likely to be replaced at increasing detergent concentrations, as directly seen by nMS (Figure 1 c).

**Figure 1:**
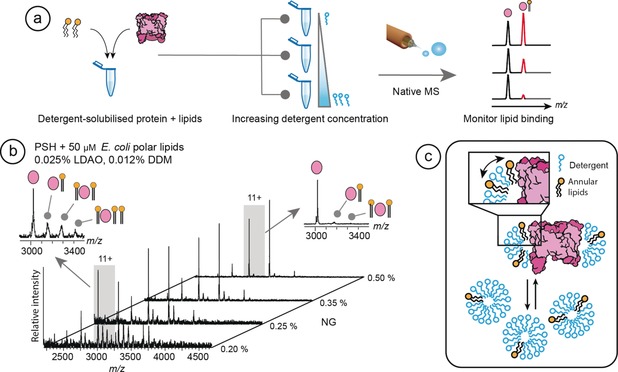
Detergents compete with annular lipids for interactions with PSH. a) Overview of the MS strategy employed. A solution of detergent‐solubilized protein with added lipids of interest is divided into multiple aliquots and supplemented with increasing amounts of detergent. Analysis by nMS shows a reduction of the lipid adduct peaks as a function of detergent concentration, revealing competition between lipids and detergent for binding to the protein. b) Addition of 50 μm *E. coli* polar lipids to PSH results in the formation of multiple lipid adducts per charge state. The 11+ charge state with lipid adducts in the presence of 0.2 % and 0.5 % NG is shown (inserts left and right, respectively). Stepwise increase of NG concentration effectively removes all bound lipids. c) Schematic representation of the competition between lipids bound at annular positions on the protein, in equilibrium with detergent molecules. The addition of excess detergent micelles dilutes the lipids, reducing binding.

The second question is whether the exchange between bound lipids and detergent molecules could be used more generally as an indicator for non‐annular, specific lipid interactions. To investigate whether we can distinguish lipids based on their exchange rates, we selected the leucine transporter LeuT from *Aquifex aeolicus*, which can be purified as a dimer with bound CDL and PG.^11^ Incubation for 16 hours in 2 % NG was found to remove the co‐purified lipids and release monomeric protein as shown previously (Figure 2 a).^11^ These findings suggest that the lipids required for dimerization are retained when the protein is solubilized in detergent, and can only be removed by prolonged incubation in particularly harsh detergent conditions (Figure 2 b).

**Figure 2:**
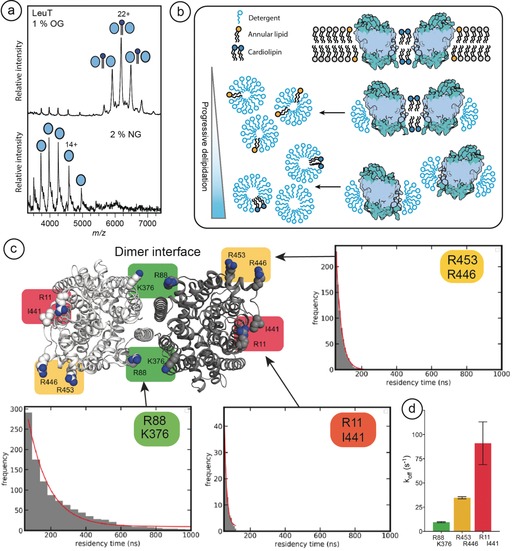
CDL exhibits extended residency times at the interface of the LeuT dimer compared to annular sites. a) nMS of LeuT shows peaks indicating in mass a CDL‐mediated dimer. Incubation in 2 % NG abolishes the dimers, and lipid‐free monomers are instead detected. b) Schematic to show that LeuT forms a native lipid‐mediated dimer in the membrane. Delipidation readily removes annular lipids around the transmembrane region, whereas removal of non‐annular lipids at the LeuT interface requires a high concentration of the detergent NG (see reference 11. c) Three CDL‐binding sites on each protomer were identified in MD simulations of LeuT in a lipid bilayer. Residency times and d) *k*_off_ rates of CDL molecules bound to all three sites were computed. CDL bound to residues R88/K376 at the dimer interface exhibited the slowest *k*_off_ and residency times up to 1000 ns, while CDL bound to the R453/R446 and the R11/I441 site showed greater than 3‐fold faster *k*_off_ rates and no lipid residency times over 200 ns. Near‐identical *k*_off_ rates were observed for both halves of the dimer (Supporting Information, Figure S2).

To investigate this possibility, we performed coarse‐grained molecular dynamics (CG‐MD) simulations of LeuT in a mixed lipid bilayer and extracted the residency times and *k*_off_ values (based on reference 15 for CDL binding to the protein). Three distinct sites on LeuT were defined based on previously generated free energy landscapes of CDL–LeuT interaction,^16^ including a site with very little observed specificity (R11/I441). For simplicity, we chose to represent each site with a pair of flanking residues (Figure 2 c and Supporting Information, Figure S2). CDL bound to the R11/I441 sites remote from the dimer interface displayed very short residency times and *k*_off_ rates greater than 70 μs^−1^. CDL interactions at the R435/R446 site exhibited slower *k*_off_ rates of 31–34 μs^−1^, possibly due to the availability of two arginine sidechains for interactions with the negatively charged phosphate head‐groups. However, CDL bound at the site composed of R88 and K376 across the dimer interface showed markedly higher residency times with some lipids bound for as long as 800 to 1000 ns, and *k*_off_ rates of less than 10 μs^−1^. As a result, the interfacial binding sites were occupied almost constantly over the course of our simulation. These findings suggest that the CDL bound to specific binding sites formed by the dimer interface exhibit a high resistance to the detergent competition. Lipids that detach more frequently, typically not interfacial lipids, have a higher probability of being displaced from the protein by detergent.

Having established that annular and non‐annular lipids can differ in their exchange with detergent, we asked whether we can use solution competition to identify lipid interactions involving non‐annular binding sites. We selected the 57 kDa *E. coli* lipid flippase MurJ, a monomeric 14‐helix integral membrane protein. MurJ catalyzes the transport of lipid‐II, an essential precursor for the synthesis of cell wall peptidoglycans in bacteria, making it an important target for antibiotics.^17^ The structural basis for MurJ flippase activity is only partially understood, primarily because the large, flexible lipid‐II substrate is not resolved in crystal structures.^18^ We have previously shown that substrate binding to MurJ is inhibited by CDL, which blocks the lipid‐II binding site.^19^ nMS analysis of MurJ extracted from *E. coli* revealed a sub‐population with a single co‐purified CDL that is retained under a variety of detergent conditions (Supporting Information, Figure S3). This led us to speculate whether CDL or lipid‐II, upon binding becomes isolated from the surrounding lipid or detergent environment. To investigate this question, we carried out analogous experiments to those performed for LeuT and PSH. We added 16:0–18:1 PE (POPE), the most abundant *E. coli* lipid and not a MurJ substrate, to the protein in 0.05 % LDAO. nMS shows binding of multiple POPE molecules per protein (Figure 3 a). The concentration of NG was then increased in a stepwise fashion and the effect on lipid binding monitored. We find that NG effectively competes with POPE for binding to the protein, reducing the number and intensity of lipid adducts in a concentration‐dependent manner (Figure 3 a). Using POPE binding to identify the most stringent competition conditions, we identified 0.5 % octylglucoside (OG) as the most efficient detergent to disrupt lipid binding to MurJ (Supporting Information, Figure S4). The observation that in this case, OG removes bound lipids more readily than NG suggests that delipidation abilities of different detergents may vary between protein systems.

**Figure 3:**
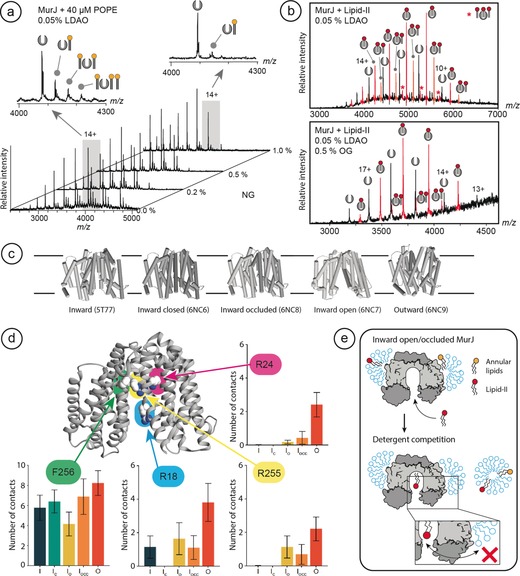
The lipid flippase MurJ binds a single lipid‐II that does not exchange with detergent. a) Lipid adducts formed by the addition of PE to MurJ are readily exchanged by increasing the concentration of NG. The 14+ charge state of MurJ in 0.05 % LDAO with lipid adducts in the presence of 0 % and 1 % NG is shown (left and right inserts, respectively) b) The addition of lipid‐II to detergent‐solubilized MurJ results in binding of one to three lipid molecules per monomer. Increasing the concentration of OG increases the average charge of MurJ but has no pronounced effect on the binding of one lipid‐II, while all additional bound lipids are removed. c) The X‐ray crystal structures of *Th. africanus* MurJ in the inward, inward‐open, inward‐occluded, inward‐closed, and outward states. d) Positions of F256 (outside), R18 (active site gate), and R24 and R255 (active site) in MurJ exhibit different accessibilities for lipid substrates depending on the transport state of the protein. Monitoring contacts between UDP acyl chains and key residues in MurJ in a PE bilayer over time reveals R18, R24, and R255 in the active site are sporadically accessed by the lipid substrate in the inward state but are exposed in the outward state. e) Schematic to show how MurJ can bind multiple lipids that exchange differently with the surrounding detergent, with a single lipid‐II exhibiting much slower *k*_off_ rates due to its protected location in the active site.

Next, we added the natural substrate lipid‐II in 2‐fold excess to MurJ in 0.05 % LDAO. From the mass spectrum we observed apo protein and protein with lipid‐II adducts, the dominant species being assigned to MurJ:(lipid‐II)_1_ and MurJ:(lipid‐II)_2_ (Figure 3 b). Stripping LDAO from a desolvated protein reduces its charge.^20^ We found that the addition of a 20‐fold excess of OG mitigates this effect, likely by diluting LDAO in mixed micelles. This results in a shift to higher charge states, which should additionally reduce lipid binding.^21^ However, while OG completely removed the second, and low‐intensity third, lipid adducts, there was no pronounced effect on the MurJ:(lipid‐II)_1_ complex compared to the spectrum recorded in 0.05 % LDAO only (Figure 3 b). This observation suggests that a single lipid‐II occupies a binding site on the MurJ monomer with a particularly slow *k*_off_ rate and is less prone to being replaced by detergent. Additional lipid‐II molecules, as well as PE moieties, can be readily substituted by detergent.

To understand why lipid‐II binds to MurJ but does not exchange with the surrounding detergent, we turned to the recent high‐resolution X‐ray crystal structures of the closely related MurJ from *Thermosipho africanus* in its inward‐open, inward‐occluded, inward‐closed, and outward states (Figure 3 c).^18^, ^22^ The substrate‐binding site is located in a deep grove between two lobes of the protein, which led us to ask whether this site is accessible to membrane lipids and detergents. We performed CG‐MD simulations of all five MurJ states in PE bilayers supplemented with a small number of undecaprenyl‐pyrophosphate (UDP) molecules, the lipid stem of lipid‐II. In these simulations, we monitored the contacts between UDP and four sites on the protein identified as important for UDP binding in the original structural studies:^18^ F256, located in the minor groove on the large lobe of the protein, R18, which is located at the gate between the membrane and the central cavity, and R24 and R255 at the bottom of the substrate‐binding site (Figure 3 d). We observed a comparably high number of contacts between UDP and F256 in all transport states, consistent with its membrane‐facing location. For R18, R24, and R255, which are conserved in *E. coli* MurJ and essential for substrate binding,^22^, ^23^ we found the highest number of contacts with UDP in the outward state. In this state, the substrate‐binding site is too shallow to accommodate the substrate, making binding unlikely.^18^, ^22^ In the inward‐occluded and inward‐open states, we observed sporadic contacts between the acyl chains of UDP and all three arginine residues, suggesting that the active site is highly protected but remains occasionally accessible to the substrate. Strikingly, all three key residues are completely inaccessible in the inward‐closed conformation, showing that the active site can be completely isolated from the membrane.

Together, these MS and MD data show therefore that while several lipids can readily bind to MurJ, only one lipid‐II adduct does not readily exchange with the detergent, indicative of binding with a slower *k*_off_ rate than the other lipids. Since lipid‐II is the natural substrate of MurJ, we explored the accessibility of the active site in MD simulations using a derivative of lipid‐II and found that a substrate can enter the active site most readily in the inward‐facing conformation partially isolated from the surrounding membrane. Excess lipid‐II, as well as other non‐specifically bound *E. coli* lipids, can be exchanged with detergent, suggesting binding in annular positions (Figure 3 e).

In summary, we have demonstrated a simple MS‐based strategy to distinguish annular from non‐annular lipids based on their ability to exchange with detergent in solution. For PSH, we showed that annular lipids could be exchanged readily for detergent with no distinction between the various lipids tested. Supporting our observations with MD simulations, we then showed that in LeuT non‐specifically bound lipids exchange more rapidly with the surrounding detergent than interfacial CDL, which exhibits slow *k*_off_ rates, and are less likely to exchange with detergent. Using this detergent‐competition assay, we were able to distinguish annular lipids from a single lipid‐II molecule bound to MurJ when the active site is transiently accessible. We believe that our approach may provide valuable insights into the distinction of annular and non‐annular lipids that modulate the structure and function of membrane proteins.

## Conflict of interest

The authors declare no conflict of interest.

## Supporting information

As a service to our authors and readers, this journal provides supporting information supplied by the authors. Such materials are peer reviewed and may be re‐organized for online delivery, but are not copy‐edited or typeset. Technical support issues arising from supporting information (other than missing files) should be addressed to the authors.

Supplementary
