# Supplementary material for: A Mass‐Spectrometry‐Based Approach to Distinguish Annular and Specific Lipid Binding to Membrane Proteins
Source: Angew Chem Int Ed Engl. 2020 Jan 29;59(9):3523–8. doi: 10.1002/anie.201914411 (PMC7065234; doi:10.1002/anie.201914411)
Supplement: Supplementary file 1 — Supplementary [file ANIE-59-3523-s001.pdf]

Angewandte Chemie
Eine Zeitschrift der Gesellschaft Deutscher Chemiker

Supporting Information

A Mass-Spectrometry-Based Approach to Distinguish Annular and Specific Lipid Binding to Membrane Proteins

Jani Reddy Bolla, Robin A. Corey, Cagla Sahin, Joseph Gault, Alissa Hummer, Jonathan T. S. Hopper, David P. Lane, David Drew, Timothy M. Allison, Phillip J. Stansfeld, Carol V. Robinson,* and Michael Landreh**

anie_201914411_sm_miscellaneous_information.pdf

Experimental Methods

Protein expression and MS analysis

LeuT, PSH and MurJ were expressed in *E. coli* and purified as described previously.[1–3] All detergents were purchased from Anatrace (Maumee, OH, USA). Phospholipids were purchased from Avanti Polar Lipids (Alabaster, AL, USA). Lipid-II was purchased from the BaCWAN Facility at University of Warwick, UK.

Purified proteins were exchanged into 0.2% NG / 0.025% LDAO (PSH), 0.05% LDAO (MurJ) or 1% octyl glucoside (OG) (LeuT) and stored at -80 °C. Immediately prior to MS analysis, proteins were exchanged into MS buffer (200 mM ammonium acetate, pH 7.5) supplemented with the appropriate detergents using Biospin6 columns (BioRad).

Phospholipids were purchased from Avanti Polar Lipids Inc (USA) and 400 µM stocks were prepared as described,[4] with the exception of *E. coli* polar lipids which were solubilized at a concentration of 20 mg/mL in H_2O supplemented with 10% DDM.

For detergent competition experiments, 5 µL lipid stocks were added to 10 µL protein in ammonium acetate to make 15 µL protein/lipid stock solutions. 1 µL of detergent in H_2O at concentrations between 0 and 4 % (NG) or 0 and 0.4% (DDM) were then added to 3 µL of the protein/lipid stock to produce protein solutions with fixed lipid concentrations of 50 µM (*E. coli* polar lipids) or 40 µM 1-Palmitoyl-2-oleoyl-sn- glycero-3-phosphoethanolamine (POPE 16:0/18:1(9z)) and final detergent concentrations between 0 and 1% (NG) or 0 and 0.1 % (DDM). Mass spectra were recorded immediately after addition of lipids and secondary detergents (see Table S1), with incubation times ranging from 1 to 10 minutes, during which samples were stored on ice. Delipidation of LeuT was carried out as described.[3] Briefly, the protein was exchanged from 1% OG into 2% NG using size exclusion chromatography, followed by 16h incubation at 4 C to remove all co-purified lipids.Samples were introduced into the mass spectrometer using gold-coated borosilicate capillaries produced in-house. Mass spectra were acquired on a Waters LCT time-of-flight

mass spectrometer (MS Vision) modified for analysis of intact protein complexes[5], and on a Q Exactive EMR or an UHMR Orbitrap mass spectrometer (Thermo)[6,7]. Settings on the Q Exactive EMR were: capillary voltage 1.2 kV, S-lens RF 100%, collisional activation in the HCD cell 100–200 V, argon UHV pressure 1.12 × 10^{-9} mbar, temperature 60 °C, resolution of the instrument 17,500 at *m/z* = 200 (a transient time of 64 ms) and ion transfer optics (injection flatapole, inter-flatapole lens, bent flatapole, transfer multipole: 8, 7, 6 and 4 V, respectively). Settings on the Q Exactive UHMR were: capillary voltage 1.2 kV, S-lens RF 100%, quadrupole selection from 2,000 to 20,000 *m/z* range, collisional activation in the HCD cell 100–200 V nitrogen, UHV pressure 5-9 x 10^{-10} mbar, temperature 60 °C, resolution of the instrument 12,500 at *m/z* = 400 (a transient time of 64 ms) and ion transfer optics (injection flatapole, inter-flatapole lens, bent flatapole, transfer multipole: 6, 5, 4 and 0 V, respectively). Settings for the LCT ToF were source pressure 1 mbar, capillary voltage 1.5 kV, cone voltage 170 V, extraction cone 4V, and the transfer lens 1.5 kV. The MS data for PSH and LeuT recorded on the Time-of-Flight MS were analysed using the MassLynx 4.1 (Waters) and the Orbitrap data for MurJ using the Xcalibur (Thermo) software packages. Protein structures were visualized using Chimera 1.13.1[8].

MD Simulations

To model the kinetics of LeuT-CDL, we built the crystal dimer of PDB 2A65 (as per reference [9]) into a membrane composed of 95% POPE and 5% cardiolipin (CDL). The protein was described using the Martini open beta 3.0.b.3.2.[10,11] Additional bonds of 500 kJ mol^{-1} nm^{-2} were applied between protein backbone beads within 1 nm. For modelling undecaprenyl pyrophosphate (UDP) interactions, for each of the MurJ PDB entries 5T77, 6NC6, 6NC7, 6NC8 and 6NC9, the protein was described using Martini 2.2[10,11] with additional stability imposed using ElNeDyn.[12] Four copies of each protein were built into a single POPE membrane (ca. 23 x 23 x 10 nm) with 24 copies of the UDP molecule in a -2 charge state (parameters to be published separately). Positional restraints on a single backbone bead kept each monomer from interacting with each other. Both systems were built using *insane*[13] and solvated with Martini waters and ions to a neutral charge. Systems were minimized using the steepest descents methods, then run for 1 ns with 5 fs timesteps and 100 ns with 20 fs timesteps, both in the NPT ensemble, with velocity-rescale temperature coupling at 323 K[14] and semi-isotropic Berendsen pressure coupling[15] at 1 bar. Electrostatics were described using the reaction field method, with a

cut-off of 1.1 nm using a potential shift modifier, and van der Waals interactions were shifted from 0.9-1.1 nm. Following this, the systems were simulated over 4 repeats of 15-18 µs (LeuT) or 1 repeat of 7-8 µs (MurJ), using velocity-rescale temperature coupling at 323 K and a semi-isotropic Parrinello-Rahman barostat.[16] All simulations were run using Gromacs 2019.[17] Images were produced using VMD [18] and data were plotted in Prism 7 (GraphPad).

For modelling the LeuT kinetics, we followed an approach described previously.[19] Minimum distance analyses were run between each of the residues shown in Figure 2c and each CDL residue in the system. These data could then be plotted to follow individual residue-CDL interactions (for example, see Figure S2a). Sites were defined on the surface of LeuT based on a previously-generated free energy landscape for LeuT-CDL interaction.[20] This landscape suggested two small CDL binding sites per LeuT protomer, one on and one distant from the dimer interface. We used these two sites, as well as a region where no significant CDL binding was observed, for the analyses here. For simplicity, each site was probed based on interaction with two key residues on the flanking edge of the site. LeuT-CDL binding events were determined based on continuous time of a CDL molecule within a 0.8 nm cutoff of either of these two residues. Note that if a CDL molecule unbound and rebound within 1 ns it was considered as a continuous interaction. Cumulative CDL residencies were binned and plotted (see Figure 2c and Figure S2b), with binding events even shorter than 25 ns considered non-specific and disregarded. The binned data were fitted to a single exponential (Figure S2b; red line) to provide a k_{off} of the CDL interaction.

For the MurJ-UDP binding analyses, contact occupancies (as a fraction of total simulation time spent within 0.8 nm) were calculated between the UDP molecule and the specified residue, for each of the 4 proteins in the system. These residues were highlighted in the original structural studies as important for UDP binding.

Supplementary References

[1] X. Li, S. Dang, C. Yan, X. Gong, J. Wang, Y. Shi, *Nature* **2013**, *493*, 56–61.

[2] J. R. Bolla, J. B. Sauer, D. Wu, S. Mehmood, T. M. Allison, C. V. Robinson, *Nat. Chem.* **2018**, *10*, 363–371.

[3] K. Gupta, J. A. C. Donlan, J. T. S. Hopper, P. Uzdavinys, M. Landreh, W. B. Struwe, D. Drew, A. J. Baldwin, P. J. Stansfeld, C. V. Robinson, *Nature* **2017**, *541*, 421–424.

[4] A. Laganowsky, E. Reading, T. M. Allison, M. B. Ulmschneider, M. T. Degiacomi, A. J. Baldwin, C. V Robinson, *Nature* **2014**, *510*, 172–175.

[5] F. Sobott, H. Hernández, M. G. McCammon, M. A. Tito, C. V. Robinson, *Anal. Chem.* **2002**, *74*, 1402–1407.

[6] J. Gault, J. A. C. Donlan, I. Liko, J. T. S. Hopper, K. Gupta, N. G. Housden, W. B. Struwe, M. T. Marty, T. Mize, C. Bechara, et al., *Nat. Methods* **2016**, *13*, 333–336.

[7] M. Van De Waterbeemd, K. L. Fort, D. Boll, M. Reinhardt-Szyba, A. Routh, A. Makarov, A. J. R. Heck, *Nat. Methods* **2017**, *14*, 283–286.

[8] E. F. Pettersen, T. D. Goddard, C. C. Huang, G. S. Couch, D. M. Greenblatt, E. C. Meng, T. E. Ferrin, *J Comput Chem* **2004**, *25*, 1605–1612.

[9] K. Gupta, J. A. Donlan, J. T. Hopper, P. Uzdavinys, M. Landreh, W. B. Struwe, D. Drew, A. J. Baldwin, P. J. Stansfeld, C. V Robinson, *Nature* **2017**, *541*, 421–424.

[10] S. J. Marrink, H. J. Risselada, S. Yefimov, D. P. Tieleman, A. H. De Vries, *J. Phys. Chem. B* **2007**, *111*, 7812–7824.

[11] L. Monticelli, S. K. Kandasamy, X. Periole, R. G. Larson, D. P. Tieleman, S. J. Marrink, *J. Chem. Theory Comput.* **2008**, *4*, 819–834.

[12] X. Periole, M. Cavalli, S. J. Marrink, M. A. Ceruso, *J. Chem. Theory Comput.* **2009**, *5*, 2531–2543.

[13] T. A. Wassenaar, H. I. Ingólfsson, R. A. Böckmann, D. P. Tieleman, S. J. Marrink, *J. Chem. Theory Comput.* **2015**, *11*, 2144–2155.

[14] G. Bussi, D. Donadio, M. Parrinello, *J. Chem. Phys.* **2007**, *126*, DOI 10.1063/1.2408420.

[15] H. J. C. Berendsen, J. P. M. Postma, W. F. van Gunsteren, a DiNola, J. R. Haak, *J. Chem. Phys.* **1984**, *81*, 3684–3690.

[16] M. Parrinello, *J. Appl. Phys.* **1981**, *52*, 7182.

[17] D. Van Der Spoel, E. Lindahl, B. Hess, G. Groenhof, A. E. Mark, H. J. C. Berendsen, *J. Comput. Chem.* **2005**, *26*, 1701–1718.

[18] W. Humphrey, A. Dalke, K. Schulten, *J. Mol. Graph.* **1996**, *14*, 33–38.

[19] R. A. Corey, E. Pyle, W. J. Allen, D. W. Watkins, M. Casiraghi, B. Miroux, I. Arechaga, A. Politis, I. Collinson, *Proc. Natl. Acad. Sci. U. S. A.* **2018**, *115*, 7967–7972.

[20] R. A. Corey, O. N. Vickery, M. S. P. Sansom, P. J. Stansfeld, *J. Chem. Theory Comput.* **2019**, *15*, 5727–5736.

[21] J. Gault, D. Lianoudaki, M. Kaldmäe, N. Kronqvist, A. Rising, J. Johansson, B. Lohkamp, S. Laín, T. M. Allison, D. P. Lane, et al., *J. Phys. Chem. Lett.* **2018**, *9*, 4082–4086.

Supplementary Table 1. Detergents used for extraction, MS analysis, and lipid competition experiments of PSH, LeuT, and MurJ. Detergents exchanged via size exclusion chromatography (SEC) are indicated as primary detergents, detergents added directly to the electrospray solution are designated as secondary detergents. All concentrations are given as final percentages.

Protein	Extraction conditions	Primary MS detergent (exchanged using SEC)	Secondary MS detergent (added directly)	Final MS detergent concentrations
PSH	2% (w/v) n-nonyl-β-D-maltopyranoside (DM) 6x CMC	0.2% β-nonyl glucoside (NG), 1x CMC, 0.025% Lauryldimethylamine oxide (LDAO), 1x CMC)	0.012% DDM (1x CMC)* and<br>0 - 0.5% NG	0.025% LDAO<br>0.012% DDM*<br>0.2 – 0.5% NG
LeuT	2% n-dodecyl-β-D-Maltopyranoside (DDM) 166x CMC	1 % n-Octyl-β-D-glucopyranoside (OG), 2x CMC	-	1 % OG
		1 % OG, 2x CMC followed by<br>2 % NG, 10x CMC	-	2 % NG
MurJ	2% DDM (166x CMC)	0.05% LDAO (2x CMC)	0 - 1% NG, 0 - 5x CMC	0.05% LDAO<br>0 – 1% NG
			0.5% sucrose monodecanoate (SM), 31x CMC	0.05% LDAO<br>0.5% SM
			0.5% octyl glucose neopentyl glycol (OGNG), 8.6x CMC	0.05% LDAO<br>0.5% OGNG
			0.5% OG, 0.5x CMC	0.05% LDAO<br>0.5% OG
			0.5% NG, 2.5x CMC	0.05% LDAO<br>0.5% NG
			0.5% LDAO, 2x CMC	0.5% LDAO
			0.5% DDM, 41x CMC	0.05% LDAO<br>0.5% DDM
			0.5% 5-cyclohexyl-1-hexyl-β-D-maltopyranoside (CYMAL-5), 4x CMC	0.05% LDAO<br>0.5% CYMAL-5
			0.5% (octyl tetraethylene glycol ether (C8E4), 2x CMC	0.05% LDAO<br>0.5% C8E4
			0.5% Triton-X 100, 31x CMC	0.05% LDAO<br>0.5% TritonX100

* 1x CMC DDM was added with *E. coli* polar lipid extract

Supplementary Figures

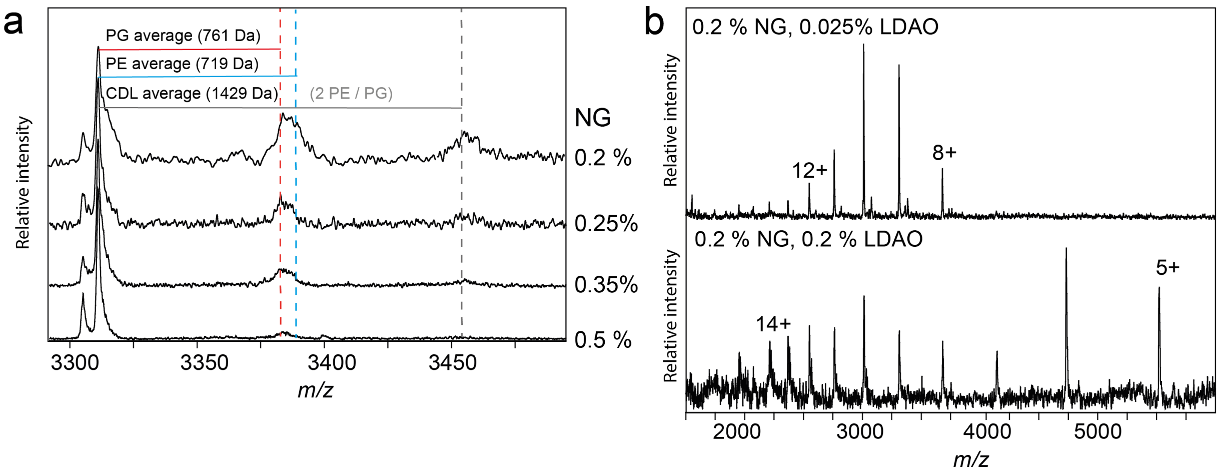

Figure S1. (a) Addition of *E. coli* polar lipid extract to PSH in 0.2% NG / 0.025 % LDAO results in binding of the main lipid species PE, PG, and CDL, as indicated by the broad adducts peaks that correspond to the average masses for each lipid species. Increasing the NG concentration from 0.2% to 0.5 % removes all different lipid adducts with no notable preference for a specific lipid type. It should be noted that peak splitting due to a 58 Da adduct was consistently observed for PSH, which did however not appear to affect lipid binding. (b) Native MS of PSH in LDAO reveals that increasing the detergent concentration from 0.025% to 0.2% reduces signal intensity and results in the appearance of lower and higher charge states of PSH, indicating unfolding. It should be noted that in addition to the appearance of higher charge states, we also observe strong charge reduction. This is due to the increased concentration of LDAO concentration, which charge-reduces proteins in a concentration-dependent manner.[21]

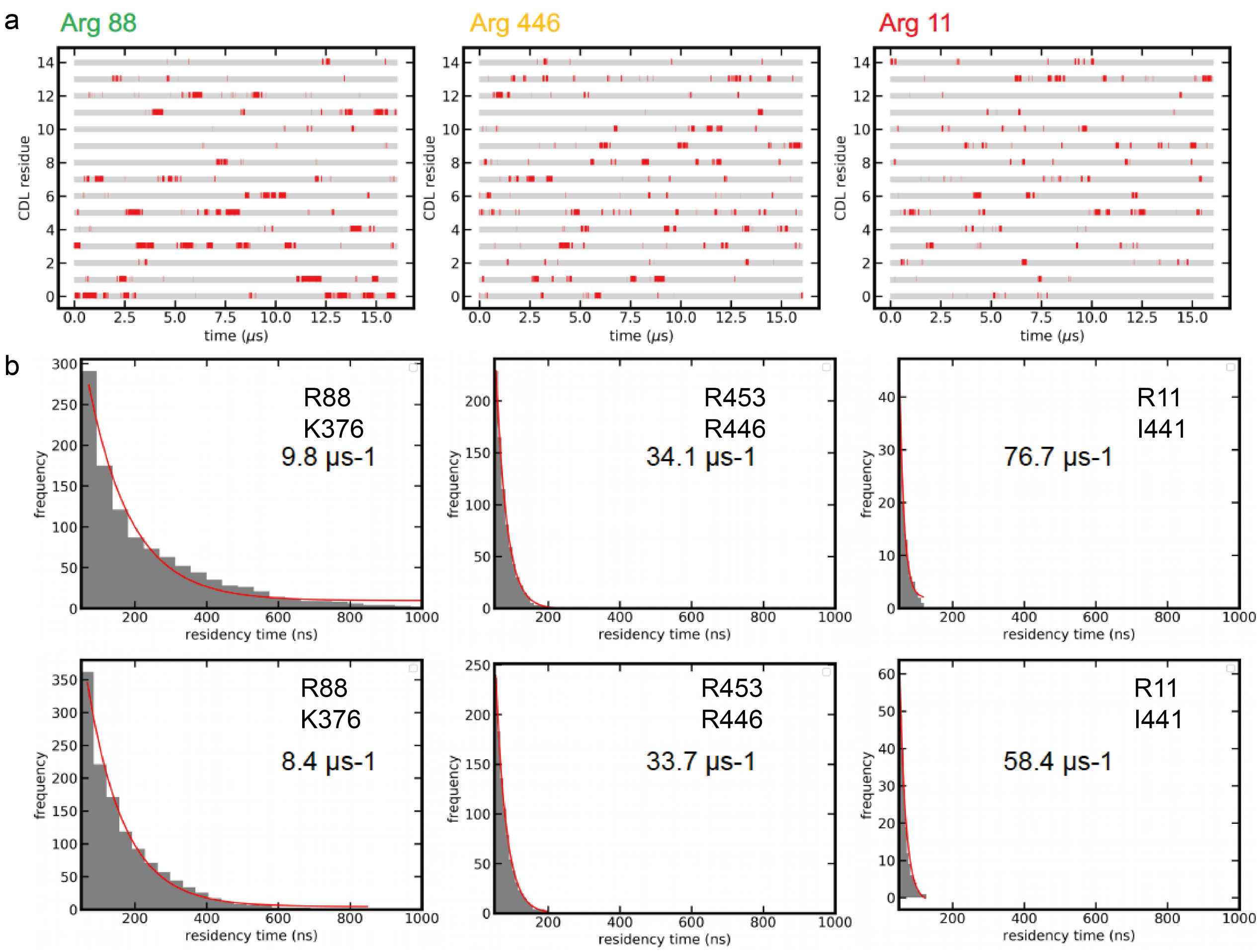

Figure S2. (a) Contacts of R88 (green), R446 (yellow), and R11 (red) with CDL molecules over the course of the simulations of LeuT in a mixed lipid bilayer. Each line indicates a specific CDL molecule in the membrane. Red bars indicate binding of the respective CDL, with bar length corresponding to residency time. (b) Distribution of the cumulative residency times for each of the three CDL binding sites on both dimer subunits. The interfacial binding site R88 / K376 displays the longest CDL residency times, with a computed k_{off} rate of 8.4 - 9.8 μs^{-1}, while the two annular sites have k_{off} rates of ca 34 – 77 μs^{-1}.

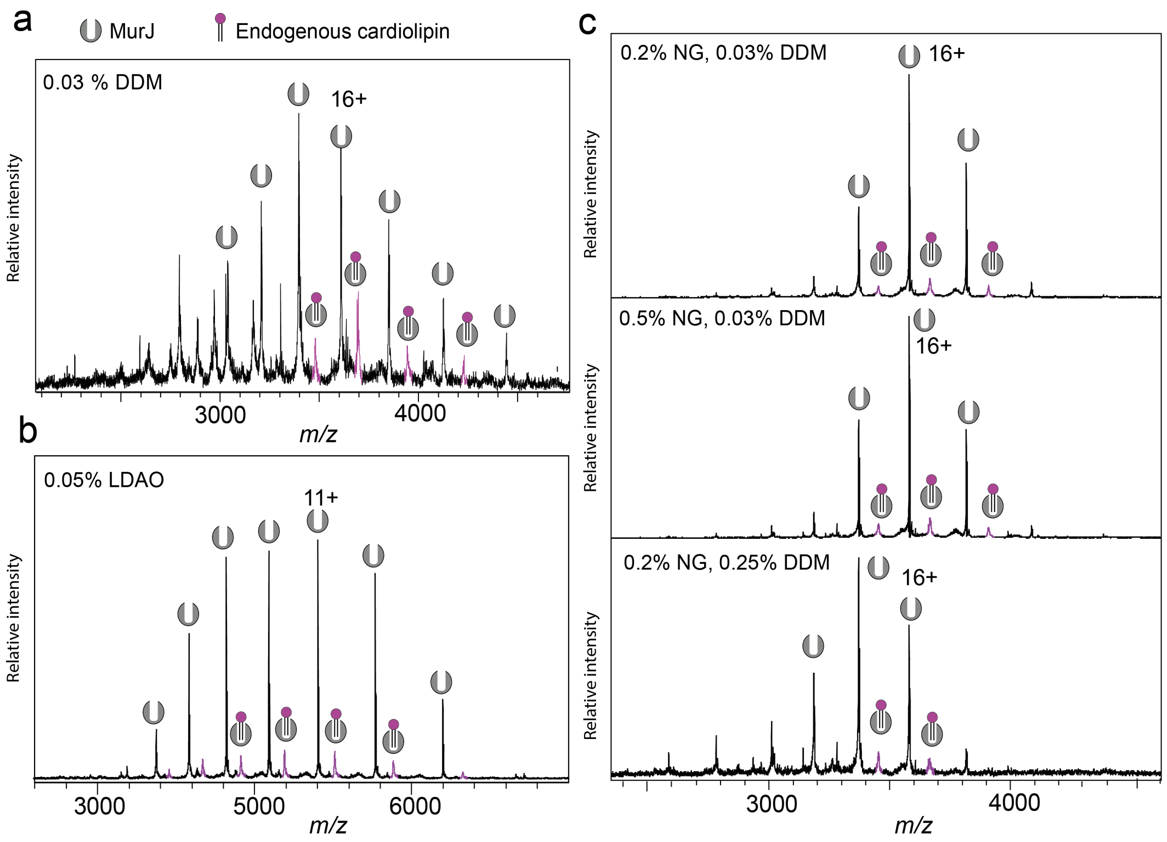

Figure S3. (a) A sub-population of *E. coli* MurJ contains an endogenous CDL adduct (marked in red) after the protein is extracted from its native bacterial membrane in 0.03% DDM. (b) The bound CDL molecule is retained when the protein is detergent-exchanged into 0.05% LDAO. (c) Addition of 0.2 % or 0.5 % NG, as well as increasing the DDM concentration to 0.25 % do not affect binding of the endogenous CDL, suggesting a highly detergent-resistant binding mode

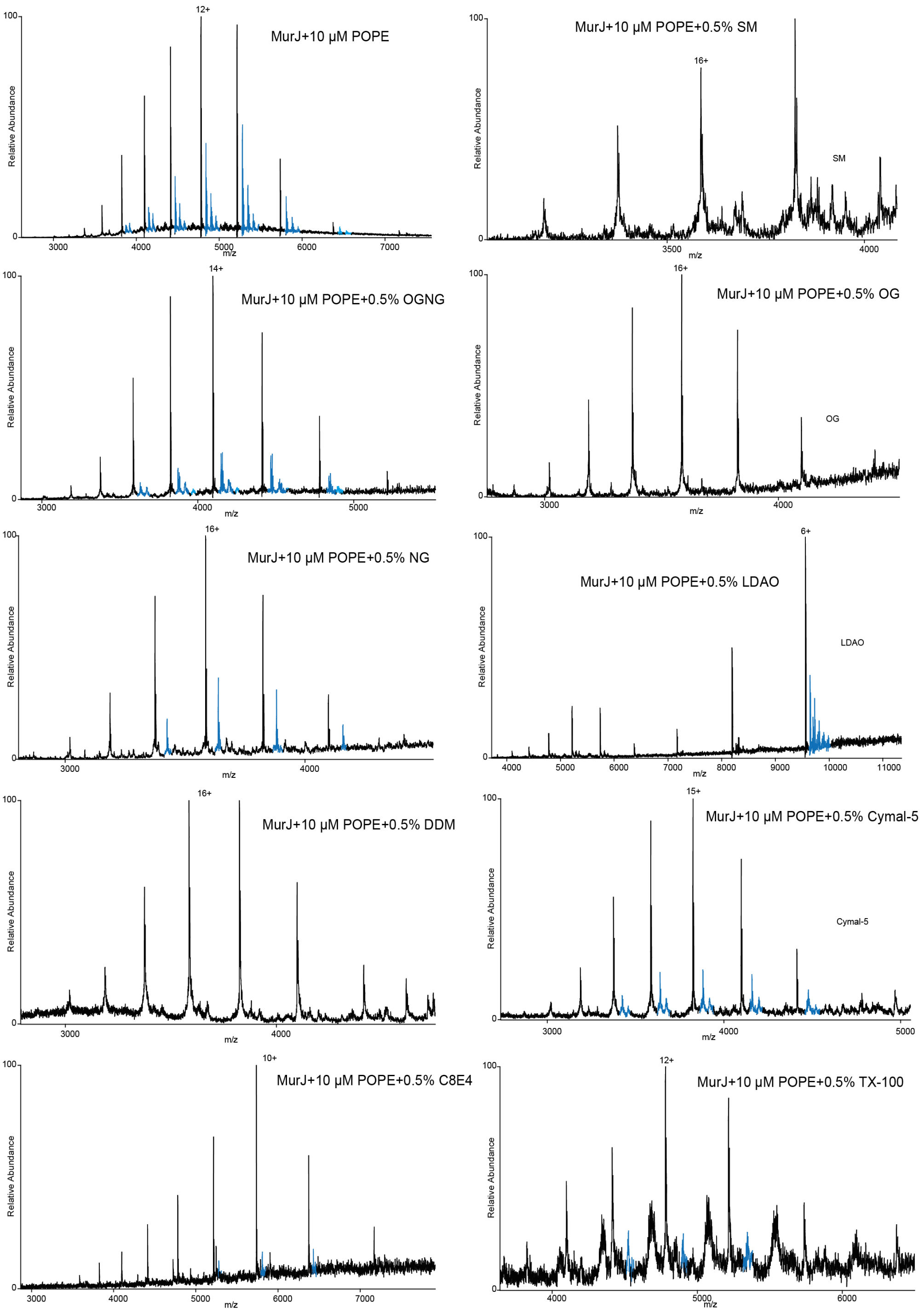

Figure S4. An MS-based detergent screen for efficient removal of annular lipids bound to MurJ. We added each detergent to the indicated final concentrations to 5 µM MurJ in 0.05 % LDAO in the presence of 10 µM POPE and monitored retention of bound POPE (blue peaks). Comparison to the spectrum of MurJ with 10 µM POPE in 0.05 % LDAO and no additional detergent (top left) reveals that 0.5 % OG is the most efficient detergent condition to remove annular POPE. A full description of each detergent condition is given in Table S1.
